# Modified cerebral small vessel disease score is associated with vascular cognitive impairment after lacunar stroke

**DOI:** 10.18632/aging.202438

**Published:** 2021-02-01

**Authors:** Nan Zhi, Lei Zhang, Yao Wang, Shuwei Bai, Jieli Geng, Ling Yu, Wenwei Cao, Lei Zhuang, Yan Zhou, Yangtai Guan

**Affiliations:** 1 Department of Neurology, Renji Hospital, School of Medicine, Shanghai Jiao Tong University, Shanghai, China; 2 Laboratory of Genetics, The Salk Institute for Biological Studies, La Jolla, CA 92037, USA; 3 Department of Radiology, Renji Hospital, School of Medicine, Shanghai Jiao Tong University, Shanghai, China

**Keywords:** small vessel disease score, cognitive impairment, executive function, visuospatial function, lacunar stroke

## Abstract

We conducted a cross-sectional study to characterize the relationship between total and modified small vessel disease (SVD) score with vascular cognitive impairment (VCI). Patients (n = 157) between the ages of 50 and 85 years old who had suffered their first lacunar infarction were analyzed prospectively. Brain magnetic resonance imaging was performed to identify SVD manifestations, which were used to calculate total or modified SVD scores. Neuropsychological assessments measured cognitive function. Spearman correlation analysis demonstrated that the total and modified SVD scores were associated with overall cognition as well as with function in the executive and visuospatial domains. The associations remained significant in linear regression after adjusting for age, sex, education and vascular risk factors. Binary logistic regression and chi-squared trend tests revealed that VCI risk increased significantly with SVD burden based on the modified SVD score. Subsequent chi-squared testing demonstrated that the VCI rate was significantly higher in patients with a modified SVD score of 5-6 than in patients without any SVD burden. Our results suggest that both the total and modified SVD scores show a negative association with cognitive function, but the modified SVD score may be better at identifying patients at high VCI risk.

## INTRODUCTION

Cerebral small vessel disease (SVD) is recognized as the leading cause of vascular cognitive impairment (VCI) in older people [1]. Early intervention is the most effective treatment to prevent cognitive decline; therefore, substantial effort has been made to stratify SVD patients at high risk of VCI. Recently, a total SVD score was proposed [2], which was shown in several prospective studies to stratify SVD patients effectively [3–5]. To calculate the total SVD score, one point is added for each of the following features : (1) microbleeds, (2) lacunes, (3) basal ganglia enlarged perivascular space (EPVS), and (4) severe periventricular or moderate to severe deep white matter hyperintensity (WMH).

A negative association between total SVD score and cognitive impairment has been demonstrated in older people [6], in patients with uncomplicated hypertension [7], and in a mixed population with either hypertension or lacunar infarction [2], but not in pure lacunar stroke patients. Usually, SVD patients with acute lacunar stroke have a relatively high SVD burden [3], so they should be monitored closely for signs of cognitive decline. We sought to discover whether VCI incidence is elevated in patients with certain total SVD scores, which might facilitate early intervention to prevent cognitive dysfunction.

At the same time, we wished to assess whether refined versions of the SVD score may be better at revealing the association between SVD burden and cognitive function. Several refinements have been proposed to the total SVD score in order to account better for individual symptoms and their severity [8–11]. One modified SVD score, for example, raises the scoring cutoff for EPVS and subdivides the points for microbleeds and WMH burden based on their number and severity [8]. We are unaware of studies on whether this modified SVD score can stratify patients based on their cognitive function.

Therefore, we investigated whether the total or modified SVD scores were associated with cognitive function and VCI risk in a homogeneous group of patients with acute lacunar stroke. Our results may improve patient screening and enable more timely intervention.

## RESULTS

### Demographic and clinical characteristics of SVD patients with acute lacunar stroke

**Figure 1:**
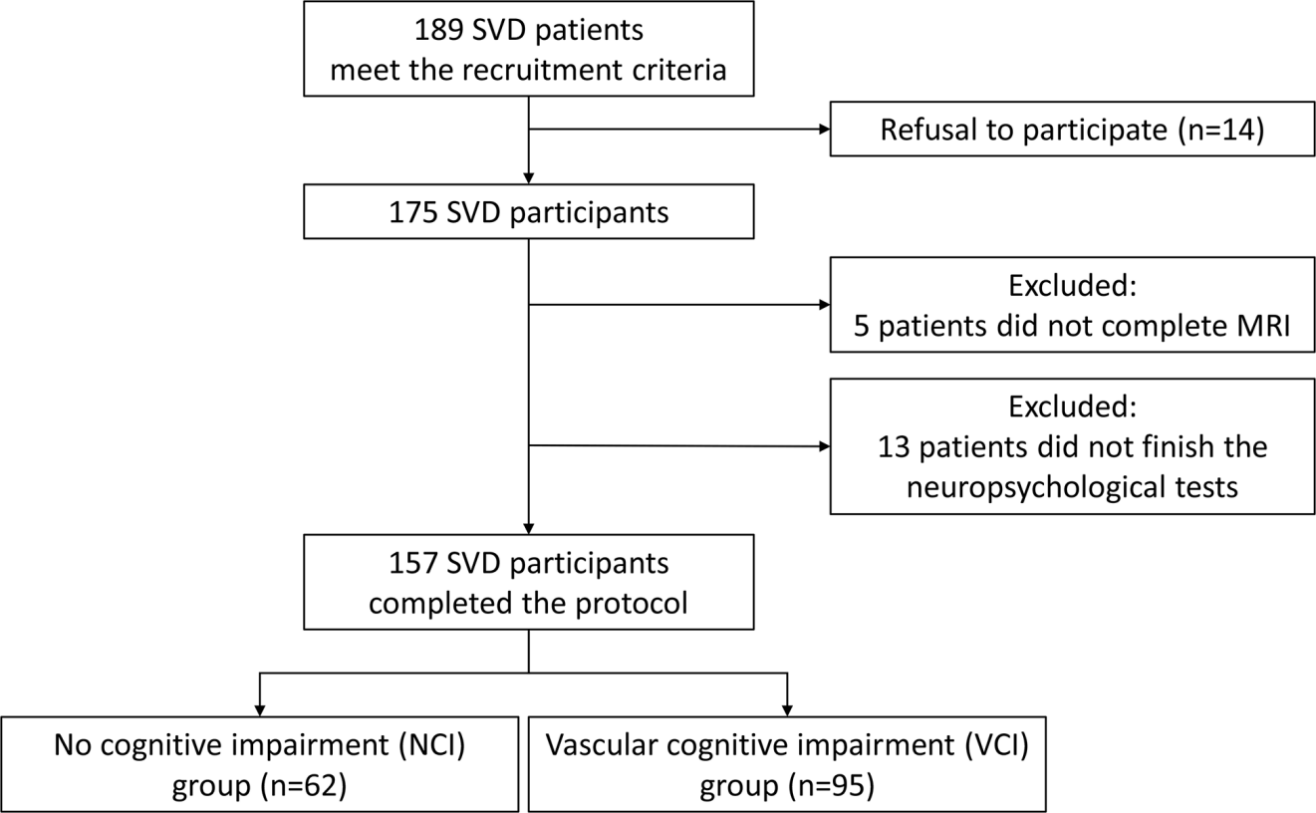
**Schematic representation of patient enrollment.** A total of 157 of 189 patients completed comprehensive evaluations, including neuroimaging and neuropsychological assessments, and were recruited into the study. Based on their performance on neuropsychological tests, patents were divided into those with vascular cognitive impairment (VCI) or those with no cognitive impairment (NCI).

In total, 189 SVD patients with acute lacunar stroke were considered eligible for the study, but 14 did not consent to participate, 5 were excluded due to incomplete brain magnetic resonance imaging (MRI), and 13 were excluded because of incomplete neuropsychological tests. A total of 157 patients were included in the final analysis (Figure 1).

Ninety-five patients were diagnosed with vascular cognitive impairment (VCI), based on their performance on neuropsychological tests. The remaining 62 patients were diagnosed with non-cognitive impairment (NCI). No significant differences were detected in age, sex, education or vascular risk factors between VCI and NCI groups (Table 1). Mean medial temporal lobe atrophy (MTA) scores also showed no significant difference between the two groups (p = 0.251); therefore, potential influence of MTA on cognitive function was not analyzed further.

**Table 1:** Demographic and clinical characteristics of SVD patients.

	**All SVD patients (n =157)**	**NCI (n =62)**	**VCI (n=95)**	**P value**
Age, years	65.27 + 7.37	65.26 + 7.31	65.27 + 7.39	0.843
Male	127 (80.9)	55 (88.7)	72 (75.8)	0.061
Education, years	10.52 + 2.90	11.2 + 3.02	10.05 + 2.73	0.089
Hypertension	111 (71.2)	48 (77.4)	63 (67.0)	0.206
Diabetes	75 (47.8)	28 (45.1)	47 (49.4)	0.494
Hypercholesterolemia	25 (16.1)	11 (18)	14 (14.9)	0.658
Current smoking	99 (63.1)	38 (61.2)	61 (64.2)	0.341
Current drinking	40 (25.5)	17 (27.4)	23 (24.2)	0.709
Mean MTA score	0.56 + 0.72	0.51 + 0.66	0.59 + 0.75	0.251
Total SVD score category				
0	27 (19.4)	12 (19.4)	15 (15.8)	
1	37 (23.6)	18 (29.0)	19 (20.0)	
2	37 (23.6)	16 (25.8)	21 (22.1)	
3	37 (23.6)	11 (17.7)	26 (27.4)	
4	19 (12.1)	5 (8.1)	14 (14.7)	
MMSE	26.12 + 2.09	27.53 + 1.34	25.21 + 2.30	0.012
Z-scores for each domain				
overall cognition	-0.44 + 0.79	0.30 + o.40	-0.93 + 0.94	0.000
executive	-1.07 + 1.03	-0.66 + 0.49	-1.72 + 1.25	0.000
memory	-0.95 + 0.81	-0.27 + 0.21	-1.40 + 0.94	0.000
language	-0.21 + 0.95	0.38 + 1.00	-0.60 + 1.04	0.000
visuospatial	0.87 (0.28,1.45)	1.16 (0.73,1.59)	0.26 (-1.44,1.58)	0.000

Values are n (%), mean + SD, or median (25^th^ quartile, 75^th^ quartile). Abbreviations: SVD, cerebral small vessel disease; NCI, no cognitive impairment; VCI, vascular cognitive impairment; SD, standard deviation; MTA, medial temporal lobe atrophy; MMSE, mini-mental state examination.

Patients were grouped according to the number of SVD manifestations present (SVD score 0-4), and comparable percentages of patients fell under each of the five scores (Table 1). For patients with scores of 1-3, the percentage of patients presenting with each type of SVD manifestation is listed in Table 2. EPVS was the most frequently occurring manifestation, while microbleeds were rarely seen.

**Table 2:** Distribution of SVD manifestations according to total SVD score.

**Manifestation**	**SVD score=1 (n=37)**	**SVD score=2 (n=37)**	**SVD score=3 (n=37)**
Asymptomatic lacune	5 (13.5)	18 (48.6)	34 (91.9)
White matter hyperintensity	9 (24.3)	24 (64.9)	34 (91.9)
Deep brain microbleeds	1 (2.7)	5 (13.5)	13 (35.1)
Basal ganglia enlarged perivascular spaces	22 (59.5)	27 (73.0)	30 (81.1)

Data are n (%). Abbreviations: SVD, cerebral small vessel disease.

The patients were further grouped according to the total SVD burden: group 1, non-burden (SVD score = 0, n = 27, 17.2%); group 2, low SVD burden (SVD score = 1 – 2, n = 74, 47.1%); and group 3, high SVD burden (SVD score = 3 – 4, n = 56, 35.7%). The demographic and clinical characteristics of each SVD group are shown in Table 3. Notably, age increased significantly with SVD burden (p = 0.002), but otherwise there were no significant differences in sex, education or vascular risk factors across the groups.

**Table 3:** Demographic and clinical characteristics of patients stratified according to SVD burden.

**Characteristic**	**Group 1^a^ (n=27)**	**Group 2^b^ (n=74)**	**Group 3^c^ (n=56)**	**P value**
Age, years	61.33 + 6.46	65.18 + 7.39	67.29 + 6.97	0.002*
Male	24 (88.9)	59 (79.7)	44 (78.6)	0.320
Education, years	10.81 + 3.37	10.53 + 2.92	10.38 + 2.66	0.813
Hypertension	18 (66.7)	52 (70.3)	42 (75)	0.407
Diabetes	6 (22.2)	32 (43.2)	17 (30.4)	0.842
Hypercholesterolemia	3 (11.1)	12 (27.3)	10 (17.8)	0.461
Current smoking	18 (66.7)	37 (50.0)	24 (42.9)	0.052
Current drinking	12 (44.4)	15 (20.3)	13 (23.2)	0.097

Values are n (%) or mean + SD. Abbreviations: SD, standard deviation. ^a^Total SVD score = 0 point. ^b^Total SVD score = 1-2 point(s). ^c^Total SVD score = 3-4 points. *P<0.05.

### Total and modified SVD scores are negatively associated with multidomain cognitive function

To determine the relationship between total SVD score and multidomain cognitive function, we carried out Spearman correlation analysis and demonstrated that SVD burden was associated with overall cognition (r_s_ = -0.253, p = 0.001) as well as with cognition involving the executive domain (r_s_ = -0.214, p = 0.007) or visuospatial domain (r_s_ = -0.254, p = 0.001), but not with memory or language domains (Table 4). The negative coefficients (r_s_) indicate that cognitive function was lower with higher SVD burden. After adjusting for age, sex, and education (Table 5, model 1), linear regression analysis further confirmed the negative associations between total SVD score and cognitive decline in overall cognition (B = -0.190, 95%CI = -0.313 to -0.067, p = 0.003) as well as in the executive domain (B = -0.268, 95%CI = -0.429 to -0.107, p = 0.001) and visuospatial domain (B = -0.296, 95%CI = -0.506 to 0.086, p = 0.006). However, no associations were detected between total SVD score and cognition in the memory domain (p = 0.088) or language domain (p = 0.406). Associations remained significant even after additionally adjusting for vascular risk factors (Table 5, model 2).

**Table 4:** Spearman correlation analyses of total or modified SVD scores with multidomain cognitive function.

**Domain**	**Total SVD score**		**Modified SVD score**	
	**r_s_**	**P value**	**r_s_**	**P value**
overall cognition	-0.253	0.001*	-0.263	0.001*
executive	-0.214	0.007*	-0.246	0.002*
memory	-0.105	0.189	-0.128	0.110
language	-0.055	0.497	-0.069	0.389
visuospatial	-0.254	0.001*	-0.202	0.011*

*P<0.05. Abbreviations: SVD, small vessel disease; r_s_, Spearman correlation coefficient.

**Table 5:** Regression analysis to identify associations between multidomain cognitive functions and total SVD score.

**Domain**		**Linear regression analysis**	**Multivariable regression analysis**	
			**Model 1^a^**	**Model 2^b^**
overall cognition	B (95%CI)	-0.185 (-0.303, -0.068)	-0.190 (-0.313, -0.067)	-0.190 (-0.315, -0.064)
	P	0.002*	0.003*	0.003*
executive	B (95%CI)	-0.249 (-0.406, -0.092)	-0.268 (-0.429, -0.107)	-0.272 (-0.435, -0.109)
	P	0.002*	0.001**	0.001**
memory	B (95%CI)	-0.084 (-0.217, 0.049)	-0.118 (-0.254, 0.018)	-0.122 (-0.260, 0.016)
	P	0.214	0.088	0.084
language	B (95%CI)	-0.093 (-0.278, 0.092)	-0.077 (-0.260, 0.106)	-0.074 (-0.259, 0.110)
	P	0.323	0.406	0.427
visuospatial	B (95%CI)	-0.315 (-0.516, -0.114)	-0.296 (-0.506, -0.086)	-0.290 (-0.504, -0.075)
	P	0.002*	0.006*	0.009*

^a^Adjusted for age, sex and educational level. ^b^Adjusted for age, sex, educational level and vascular risk factors (hypertension, hypercholesterolemia, diabetes, smoking and drinking). *P<0.05. **P<0.001 Abbreviations: B, nonstandardized coefficients; CI, confidence interval.

We next asked whether a modification of the total SVD score [8] could more accurately describe the association between SVD burden and cognitive dysfunction. Specifically, we modified the total SVD score by raising the cutoff value for basal ganglia EPVS and adjusting the scoring weighting of microbleeds and WMH according to a previous report [8]. Consistently, we observed a negative correlation of SVD burden with overall cognition (r_s_ = -0.263, p = 0.001) and with cognition in the executive domain (r_s_ = -0.246, p = 0.002) and visuospatial domain (r_s_ = -0.202, p = 0.011), but not in the memory or language domains (Table 4). The modified and total SVD scores gave similar r_s_ coefficients for the significant correlations, suggesting that the strength of those correlations did not depend on the scoring system.

### High modified SVD scores are associated with increased risk of VCI

**Figure 2:**
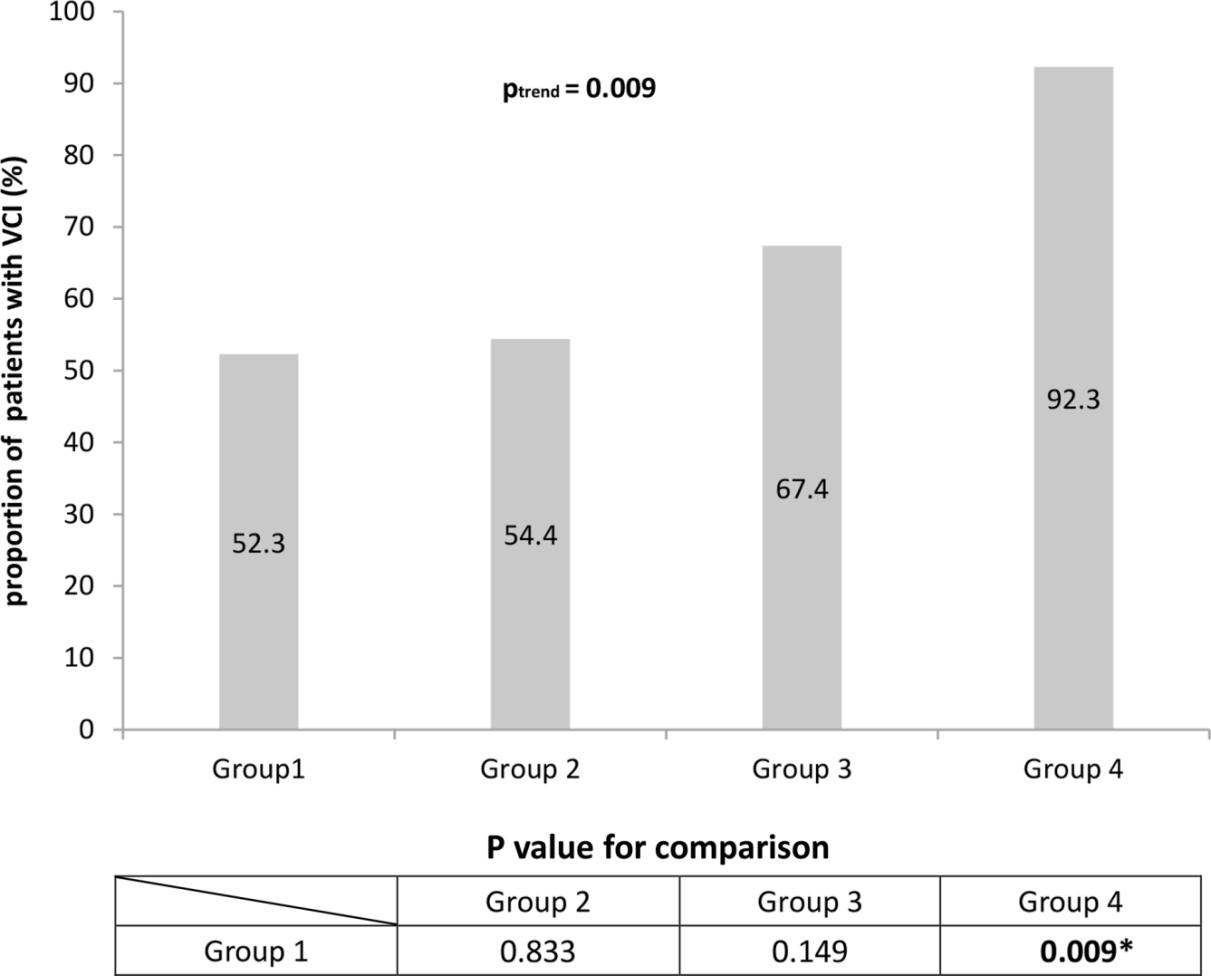
**Proportions of patients with vascular cognitive impairment (VCI) based on modified SVD score.** When patients were stratified based on the modified SVD score, the proportion of patients with VCI was significantly higher among those with high SVD burden (P = 0.009). This suggests that the modified SVD score is better at identifying patients at high risk of SVD.

Next we asked whether patients with certain total or modified SVD scores may be at greater risk of VCI. Binary logistic regression showed that risk of VCI increased with the modified SVD score (p_trend_ = 0.01, Table 6), but not the total SVD score (p_trend_ = 0.086). The chi-squared trend test gave a similar p_trend_ for the modified SVD score (Figure 2). The odds ratio calculated for risk of VCI in patients with a modified SVD score of 5-6 (group 4) was 10.957 (95%CI = 1.310 to 91.649) relative to patients with a modified score of 0 (group 1) (Table 6). Consistently, we found that the prevalence of VCI increased significantly from 52.3% in group 1 to 92.3% in group 4 (p = 0.009, Figure 2).

**Table 6:** Logistic regression analysis to assess the association of total or modified SVD score with risk of vascular cognitive impairment.

	**n**	**OR**	**95% CI**
**Total SVD score**			
Group 1^a^	15	1.00 (ref)	
Group 2^b^	40	0.941	0.388-2.283
Group 3^c^	40	2.000	0.769-5.198
P_trend_	0.086
**Modified SVD score**			
Group 1^a^	23	1.00 (ref)	
Group 2^b^	31	1.089	0.495-2.395
Group 3^c^	29	1.891	0.792-4.514
Group 4^d^	12	10.957	1.310-91.649
P_trend_		0.010*	

^a^Total or modified SVD score = 0 point. ^b^Total or modified SVD score = 1-2 point(s). ^c^Total or modified SVD score = 3-4 points. ^d^Modified SVD score = 5-6 points. *P<0.05 by trend analysis in binary logistic regression. Abbreviations: SVD, small vessel disease; OR, odds ratio; CI, confidence interval.

## DISCUSSION

In the current study, we demonstrated that the total SVD score was negatively correlated with cognitive function in patients with acute lacunar stroke. Our results are consistent with an earlier study [2] where the total SVD score was negatively associated with cognitive impairment in a heterogeneous population of patients suffering from both hypertension and lacunar stroke. Compared to an elderly population [6] and patients with hypertension [7], patients with lacunar stroke carry a higher total SVD burden [3] and are at higher risk of recurrent stroke [8]. These characteristics render patients with lacunar stroke more vulnerable to cognitive dysfunction. There is an urgent need to identify SVD patients at high risk of VCI in order to ensure timely prevention, treatment and management. Therefore, we focused our study on patients with acute lacunar stroke. Our sample contained comparable numbers of patients spanning the SVD spectrum, which may make our results more generalizable.

We examined the correlation of the original total SVD score [2] with cognitive dysfunction in our patients and with risk of VCI, and we repeated the analysis using a modified SVD score [8] that grew out of two past findings: (a) among patients who had suffered transient ischemic attack or ischemic stroke, those who scored >20 based on EPVS were at greater risk of recurrent stroke than those who scored <11; and (b) risk of recurrent stroke increased with greater burden of microbleeds and WMH. In the present patient sample, the correlation between SVD burden and cognitive dysfunction was similar under the total or modified SVD score, consistent with the two scores’ similar ability to predict recurrent stroke [8]. In contrast, we observed that the modified SVD score, but not the original total score, identified patients at high VCI risk: a modified SVD score of 5-6 was associated with significantly greater risk of VCI in our sample. These data suggest that the modified SVD score may be useful in clinical practice for identifying SVD patients at high VCI risk.

We found a negative correlation between SVD score and executive function. This finding is consistent with previous reports linking lacunar infarcts, WMH, deep microbleeds and EPVS to executive dysfunction [2] [12–16]. The frontal subcortical pathway, a key substrate in executive control networks [17–22], might also be affected by SVD manifestations and might contribute to executive dysfunction.

We also found a negative correlation of SVD score with visuospatial function. Previous studies [23, 24] showed that visuospatial dysfunction is strongly associated with white matter network disruption. Thus, WMH and microbleeds in white matter network pathways might contribute to visuospatial dysfunction after acute lacunar stroke. Such dysfunction may also reflect problems in the fronto-parietal pathway, which helps transform integrated sensory inputs into body movement during spatial tasks [25]. Increased SVD burden has been associated with reduced brain connectivity [26] and disruptions in pathways of the fronto-parieto-occipital network [27].

Since vascular disorders and Alzheimer’s disease (AD) are the most prevalent cerebral disorders in the elderly and share similar risk factors [28, 29], previous studies of SVD patients likely included patients with AD. In our study, we assessed our patients using the MTA scale [30, 31]. A cut-off score of 1.5 can sensitively detect AD patients with dementia younger than 75 years old [32, 33], and our patients, who had an average age of 65, had scores far below 1.5 (mean 0.56, range 0.00 -1.00). Thus, patients with typical AD characteristics probably did not confound our analysis of cognitive function. Nevertheless, future studies should exclude any influence of early-onset AD though comprehensive analysis such as neuroimaging to detect widespread cortical atrophy, in particular parietal lobe atrophy [34], as well as laboratory analysis of biomarkers and positron emission tomography to detect amyloid and tau aggregates.

Although this study elucidated key associations and the potential clinical relevance of a modified SVD scoring system, a few limitations are worth mentioning. Our sample was predominantly male, although we attempted to reduce potential sex bias during the statistical analysis. Our r_s_ coefficients linking SVD scores to cognitive function (between -0.2 and -0.3) were weaker than in a previous report (-0.312 and -0.438) [2], which may reflect our small, male-dominated sample. Nevertheless, we confirmed the negative association using linear regression analysis and after adjusting for sex and other confounders. In addition, the nonstandardized B regression coefficients (between -0.185 and -0.315) were consistent with those in several other studies (between -0.08 and -0.18) [6, 7, 35]. Future work with larger, sex-balanced samples should verify and expand our findings. In particular, longitudinal studies are needed to examine whether the modified SVD score can identify or even predict acute lacunar stroke patients at high risk of VCI.

## MATERIALS AND METHODS

### Participants

Consecutive SVD patients with first-ever lacunar stroke were recruited prospectively between January 2016 and January 2019 from the stroke clinic at the Department of Neurology of Renji Hospital affiliated to Shanghai Jiao Tong University School of Medicine (Renji Cerebral SVD Cohort Study, NCT01334749 at http://clinicaltrials.gov/). The current study was approved by the Ethics Committee of Renji Hospital affiliated to Shanghai Jiao Tong University School of Medicine. To be enrolled, patients had to have first-ever acute lacunar stroke as defined previously [36], at least six years of education, age between 50 and 85 years, and a modified Rankin score ≤ 3 points. Patients were excluded if they had any of the following: cardioembolic or large-vessel diseases; severe systemic or other diseases that may cause cognitive dysfunction; cortical and/or cortico-subcortical non-lacunar infarct, or WMH due to other specific causes; severe depression (17-item Hamilton Depression Rating Scale score ≥ 24); other major central neurological or psychiatric disorders; or severe claustrophobia or contraindications to MRI examination.

Lacunes were defined as sharply demarcated hyperintense lesions <20 mm on T2-weighted MRI that showed a hyperintense rim on T2-fluid attenuated inversion recovery (FLAIR) sequences [37]. Recent symptomatic small subcortical infarcts were not counted as lacunes.

Of 189 patients initially enrolled, 157 completed the following comprehensive evaluations: sociodemographic and clinical data collection, neurological and physical examination, laboratory tests, neuropsychological assessments and brain MRI. Brain MRI was conducted within 7 days (4 ± 2.34) after stroke, but neuropsychological assessments were performed 3 months after stroke in order to exclude any effects due to acute stroke.

A mean MTA score ≥1.5 for both hemispheres has shown high sensitivity in identifying AD patients younger than 75 who convert to dementia [32, 33]. Therefore patients were assessed on the MTA scale in order to assess whether AD may have affected cognitive function in our sample. MTA score was rated visually using a 5-grade rating scale from 0 (no atrophy) to 4 (severe atrophy), as described [31]. Briefly, the width of the choroidal fissure, width of the temporal horn and the height of the hippocampal formation were visually assessed in the best slice that depicted both hippocampal formations at the anterior pons. The final MTA score was calculated as the average value from both hemispheres.

### Brain MRI

All brain MRI scans were acquired on a GE Signa HDxT 3.0T MRI scanner (General Electric Medical Systems, Milwaukee, WI, USA) with a standard 8-channel head coil with foam padding. The following MRI images were obtained: 3D-fast spoiled gradient recalled (SPGR) sequence images (TR = 6.1 ms, TE = 2.8 ms, TI = 450 ms, slice thickness = 1.0 mm, gap = 0, flip angle = 15° C, FOV = 256 × 256 mm^2^, number of slices = 166), axial T2-weighted fast spin-echo sequence images (TR = 3013 ms, TE = 80 ms, FOV = 256 × 256 mm^2^, number of slices = 34), T2-FLAIR sequence images (TE = 150 ms, TR = 9075 ms, TI = 2250 ms, FOV = 256 mm^2^, number of slices = 66), and gradient recalled echo (GRE) T2-weighted sequence images (TR = 53.58 ms, TE = 23.93 ms, flip angle = 2° C, matrix = 320 × 288, FOV = 240 × 240 mm^2^, slice thickness = 2 mm, NEX = 0.7, gap = 0, slices = 72).

### Total SVD score

The total SVD score was calculated according to previous reports [2]. Briefly, one point was allocated for each of the following manifestations: lacune, severe periventricular (Fazekas score 3) or moderate-to-severe deep WMH (Fazekas score 2 or 3), deep microbleeds, or moderate-to-severe EPVS (>10) in basal ganglia. WMH were graded on the Fazekas scale [38], while periventricular and deep WMH were graded from 0 to 3 based on T2-FLAIR sequences. Microbleeds were defined as punctate (< 10 mm), homogeneous foci of low signal intensity on GRE T2*-weighted images. To evaluate the number of microbleeds, the microbleed anatomical rating scale (MARS) was employed [39]. The number of deep microbleeds was also determined, because SVD has been associated specifically with deep microbleeds that include the basal ganglia and thalamus as well as internal, external and extreme capsules [40]. EPVS was defined as round, oval or linear lesions with a smooth margin that gave the same signal as cerebrospinal fluid on MRI, i.e. low signal on T1WI, high signal on T2WI, and low signal without a hyperintense rim on the FLAIR sequence. EPVS at the level of the basal ganglia was scored as described [41] on only one slice on one side in the more affected hemisphere. Patients were stratified by EPVS burden into three score groups: <11, 11–20, or >20.

Images were independently rated by two radiologists (Yan Zhou and Yao Wang), who resolved any discrepancies through discussion. Test–retest reliability was analyzed using 15 random samples, which yielded an intra-class correlation coefficient (ICC) of 0.815 for lacune number, 0.786 for total Fezakas score, 0.792 for deep microbleed number and 0.857 for EPVS number.

Patients were divided into three groups according to their SVD score: group 1 (0 point), group 2 (1-2 points), and group 3 (3-4 points).

### Modified SVD score

The modified SVD score was computed based on the presence of the abovementioned four MRI markers, with the scoring standards adjusted based on a previous report [8]. In the present study, one point was allocated to those who had >20 EPVS, rather than to those who had >10 basal ganglia EPVS as in the previous study [2]. Burden of deep microbleeds was accounted for by assigning 1 point to patients with 1-4 microbleeds and 2 points to those with >4 microbleeds. Burden of total (periventricular and deep) WMH was accounted for by allocating 1 point to those with a moderate degree of WMH (combined score of 3 or 4) and 2 points to those with severe WMH (combined score of 5 or 6). Similarly to total SVD scoring, one point was awarded when one or more lacunes were present. As a result, the modified total score of SVD ranged from 0 to 6. Modified SVD scores were determined independently by the same two investigators who calculated total scores, and the ICC was 0.801.

Patients were divided into four groups according to their modified SVD score: group 1 (0 point), group 2 (1-2 points), group 3 (3-4 points), and group 4 (5-6 points).

### Neuropsychological assessments

The mini-mental state examination (MMSE) [42] was used to assess overall cognitive performance. Each patient also completed the following battery of neuropsychological tests to assess multiple domains: (1) executive function was assessed using the Trail-Making Test A and B [43], Stroop color-word test [44] and category Verbal Fluency Test [45]; (2) memory was assessed using the Rey Auditory Verbal Learning Test of short- and long-delay free recall [46]; (3) language was assessed using the 30 words of the Boston naming test [47]; and (4) visuospatial function was assessed using the Rey-Osterrieth Complex Figure Test [48]. The original score for each neuropsychological test was transformed to a Z score (a Z score of +1.0 corresponds to a score that is 1.0 standard deviation above the mean), and Z-scores for each domain were generated by averaging the Z-scores of the corresponding tests. Norms for Z scoring were based on the mean scores from community studies of elderly in Shanghai, China [49, 50]. Cognitive impairment was defined as a score lying 1.5 standard deviations below the mean on any neuropsychological test [51]. Patients were divided into a group with no cognitive impairment (NCI) and a group with vascular cognitive impairment (VCI) [51].

Neuropsychological assessments were conducted in a dedicated office by two experienced investigators (Ling Yu and Wenwei Cao) certified to administer neuropsychological assessments. All assessments of a given patient were carried out by one investigator in order to minimize intra-patient variability. Inter-rater reliability was assessed for the Z-scores for each domain. ICC was 0.796 for the executive domain, 0.801 for the memory domain, 0.789 for the language domain and 0.815 for the visuospatial domain. In addition, the kappa coefficient for determining VCI was 0.792.

### Statistical analysis

Differences in baseline characteristics between the NCI group and VCI group were assessed for significance using the independent *t*-test for continuous variables and chi-squared test for categorical variables (sex and vascular risk factors). Z scores on visuospatial domain were skewed, so differences were assessed using the non-parametric Mann–Whitney test. Spearman correlation analysis was used to determine associations between SVD score and multidomain cognitive functions. Simple linear regression analysis was performed with SVD score as the independent variable and Z-scores of multidomain cognitive function as dependent variables (overall cognition, executive domain, memory domain, language domain, visuospatial domain). The associations were adjusted for possible confounders using two multivariable linear regression models. In model 1, the associations between SVD score and multidomain cognitive scores were adjusted for age, sex and educational level. Model 2 adjusted for these same factors as well as potential confounding vascular risk factors (smoking, drinking, and the presence of diabetes mellitus, hypertension or hypercholesterolemia).

To determine the association of SVD burden and risk of VCI, binary logistic regression analysis was performed with total SVD score or modified SVD score as covariables, cognitive state as dependent variable (VCI or NCI), and group 1 (score of 0) as a dummy variable. To determine how the risk of VCI changed with a rise in SVD burden, trend analysis in binary logistic regression was carried out in SPSS. For validation, the linear-by-linear-association chi-squared statistic was calculated using the crosstabs routine in SPSS. The chi-squared test was performed to compare the prevalence of VCI in modified SVD score groups 2, 3 or 4 with the prevalence in group 1. SPSS 23.0 (IBM, USA) was used for all analyses. Differences were considered significant at *p* < 0.05.

## References

[1] Pantoni L. Cerebral small vessel disease: from pathogenesis and clinical characteristics to therapeutic challenges. Lancet Neurol. 2010; 9:689–701. 10.1016/S1474-4422(10)70104-6 20610345

[2] Huijts M, Duits A, van Oostenbrugge RJ, Kroon AA, de Leeuw PW, Staals J. Accumulation of MRI markers of cerebral small vessel disease is associated with decreased cognitive function. A study in first-ever lacunar stroke and hypertensive patients. Front Aging Neurosci. 2013; 5:72. 10.3389/fnagi.2013.00072 24223555 PMC3818574

[3] Staals J, Makin SD, Doubal FN, Dennis MS, Wardlaw JM. Stroke subtype, vascular risk factors, and total MRI brain small-vessel disease burden. Neurology. 2014; 83:1228–34. 10.1212/WNL.0000000000000837 25165388 PMC4180484

[4] Yakushiji Y, Charidimou A, Noguchi T, Nishihara M, Eriguchi M, Nanri Y, Kawaguchi A, Hirotsu T, Werring DJ, Hara H. Total small vessel disease score in neurologically healthy Japanese adults in the Kashima scan study. Intern Med. 2018; 57:189–96. 10.2169/internalmedicine.8393-16 29033410 PMC5820035

[5] Jiang J, Huang X, Zhang Y, Deng W, Shen F, Liu J. Total MRI burden of cerebral vessel disease correlates with the progression in patients with acute single small subcortical strokes. Brain Behav. 2019; 9:e01173. 10.1002/brb3.1173 30506998 PMC6346414

[6] Staals J, Booth T, Morris Z, Bastin ME, Gow AJ, Corley J, Redmond P, Starr JM, Deary IJ, Wardlaw JM. Total MRI load of cerebral small vessel disease and cognitive ability in older people. Neurobiol Aging. 2015; 36:2806–11. 10.1016/j.neurobiolaging.2015.06.024 26189091 PMC4706154

[7] Uiterwijk R, van Oostenbrugge RJ, Huijts M, De Leeuw PW, Kroon AA, Staals J. Total cerebral small vessel disease MRI score is associated with cognitive decline in executive function in patients with hypertension. Front Aging Neurosci. 2016; 8:301. 10.3389/fnagi.2016.00301 28018214 PMC5149514

[8] Lau KK, Li L, Schulz U, Simoni M, Chan KH, Ho SL, Cheung RT, Küker W, Mak HK, Rothwell PM. Total small vessel disease score and risk of recurrent stroke: validation in 2 large cohorts. Neurology. 2017; 88:2260–67. 10.1212/WNL.0000000000004042 28515266 PMC5567324

[9] Molad J, Kliper E, Korczyn AD, Ben Assayag E, Ben Bashat D, Shenhar-Tsarfaty S, Aizenstein O, Shopin L, Bornstein NM, Auriel E. Only white matter hyperintensities predicts post-stroke cognitive performances among cerebral small vessel disease markers: results from the TABASCO study. J Alzheimers Dis. 2017; 56:1293–99. 10.3233/JAD-160939 28157096

[10] Kim YB, Park KY, Chung PW, Kim JM, Moon HS, Youn YC. Brachial-ankle pulse wave velocity is associated with both acute and chronic cerebral small vessel disease. Atherosclerosis. 2016; 245:54–59. 10.1016/j.atherosclerosis.2015.12.006 26707255

[11] Arba F, Inzitari D, Ali M, Warach SJ, Luby M, Lees KR, and STIR/VISTA Imaging Collaboration. Small vessel disease and clinical outcomes after IV rt-PA treatment. Acta Neurol Scand. 2017; 136:72–77. 10.1111/ane.12745 28233290

[12] Carey CL, Kramer JH, Josephson SA, Mungas D, Reed BR, Schuff N, Weiner MW, Chui HC. Subcortical lacunes are associated with executive dysfunction in cognitively normal elderly. Stroke. 2008; 39:397–402. 10.1161/STROKEAHA.107.491795 18096844 PMC2443738

[13] Werring DJ, Frazer DW, Coward LJ, Losseff NA, Watt H, Cipolotti L, Brown MM, Jäger HR. Cognitive dysfunction in patients with cerebral microbleeds on T2*-weighted gradient-echo MRI. Brain. 2004; 127:2265–75. 10.1093/brain/awh253 15282216

[14] van den Heuvel DM, ten Dam VH, de Craen AJ, Admiraal-Behloul F, Olofsen H, Bollen EL, Jolles J, Murray HM, Blauw GJ, Westendorp RG, van Buchem MA. Increase in periventricular white matter hyperintensities parallels decline in mental processing speed in a non-demented elderly population. J Neurol Neurosurg Psychiatry. 2006; 77:149–53. 10.1136/jnnp.2005.070193 16421114 PMC2077562

[15] Zhu YC, Dufouil C, Soumaré A, Mazoyer B, Chabriat H, Tzourio C. High degree of dilated Virchow-Robin spaces on MRI is associated with increased risk of dementia. J Alzheimers Dis. 2010; 22:663–72. 10.3233/JAD-2010-100378 20847444

[16] Huijts M, Duits A, Staals J, Kroon AA, de Leeuw PW, van Oostenbrugge RJ. Basal ganglia enlarged perivascular spaces are linked to cognitive function in patients with cerebral small vessel disease. Curr Neurovasc Res. 2014; 11:136–41. 10.2174/1567202611666140310102248 24606607

[17] Biesbroek JM, Weaver NA, Biessels GJ. Lesion location and cognitive impact of cerebral small vessel disease. Clin Sci (Lond). 2017; 131:715–28. 10.1042/CS20160452 28385827

[18] O’Sullivan M. Imaging small vessel disease: lesion topography, networks, and cognitive deficits investigated with MRI. Stroke. 2010; 41:S154–58. 10.1161/STROKEAHA.110.595314 20876494

[19] Patel B, Lawrence AJ, Chung AW, Rich P, Mackinnon AD, Morris RG, Barrick TR, Markus HS. Cerebral microbleeds and cognition in patients with symptomatic small vessel disease. Stroke. 2013; 44:356–61. 10.1161/STROKEAHA.112.670216 23321452

[20] Mok VC, Liu T, Lam WW, Wong A, Hu X, Guo L, Chen XY, Tang WK, Wong KS, Wong S. Neuroimaging predictors of cognitive impairment in confluent white matter lesion: volumetric analyses of 99 brain regions. Dement Geriatr Cogn Disord. 2008; 25:67–73. 10.1159/000111692 18042992

[21] Liu R, Chen H, Qin R, Gu Y, Chen X, Zou J, Jiang Y, Li W, Bai F, Zhang B, Wang X, Xu Y. The altered reconfiguration pattern of brain modular architecture regulates cognitive function in cerebral small vessel disease. Front Neurol. 2019; 10:324. 10.3389/fneur.2019.00324 31024423 PMC6461194

[22] Dey AK, Stamenova V, Turner G, Black SE, Levine B. Pathoconnectomics of cognitive impairment in small vessel disease: a systematic review. Alzheimers Dement. 2016; 12:831–45. 10.1016/j.jalz.2016.01.007 26923464

[23] Kim HJ, Im K, Kwon H, Lee JM, Ye BS, Kim YJ, Cho H, Choe YS, Lee KH, Kim ST, Kim JS, Lee JH, Na DL, Seo SW. Effects of amyloid and small vessel disease on white matter network disruption. J Alzheimers Dis. 2015; 44:963–75. 10.3233/JAD-141623 25374100

[24] Price CC, Jefferson AL, Merino JG, Heilman KM, Libon DJ. Subcortical vascular dementia: integrating neuropsychological and neuroradiologic data. Neurology. 2005; 65:376–82. 10.1212/01.wnl.0000168877.06011.15 16087901 PMC2746450

[25] Whitlock JR. Posterior parietal cortex. Curr Biol. 2017; 27:R691–95. 10.1016/j.cub.2017.06.007 28743011

[26] Valenti R, Reijmer YD, Charidimou A, Boulouis G, Martinez SR, Xiong L, Fotiadis P, Jessel M, Ayres A, Riley G, Pantoni L, Edip Gurol M, Greenberg SM, Viswanathan A. Total small vessel disease burden and brain network efficiency in cerebral amyloid angiopathy. J Neurol Sci. 2017; 382:10–12. 10.1016/j.jns.2017.09.015 29110998 PMC5907925

[27] Sheorajpanday RV, Marien P, Weeren AJ, Nagels G, Saerens J, van Putten MJ, De Deyn PP. EEG in silent small vessel disease: sLORETA mapping reveals cortical sources of vascular cognitive impairment no dementia in the default mode network. J Clin Neurophysiol. 2013; 30:178–87. 10.1097/WNP.0b013e3182767d15 23545769

[28] Dichgans M, Zietemann V. Prevention of vascular cognitive impairment. Stroke. 2012; 43:3137–46. 10.1161/STROKEAHA.112.651778 22935401

[29] Traylor M, Adib-Samii P, Harold D, Dichgans M, Williams J, Lewis CM, Markus HS, and Alzheimer’s Disease Neuroimaging Initiative, and International Stroke Genetics Consortium (ISGC), and UK Young Lacunar Stroke DNA resource, and METASTROKE, and International Genomics of Alzheimer’s Project (IGAP), and investigators. Shared genetic contribution to Ischaemic Stroke and Alzheimer’s disease. Ann Neurol. 2016; 79:739–47. 10.1002/ana.24621 26913989 PMC4864940

[30] Scheltens P, Leys D, Barkhof F, Huglo D, Weinstein HC, Vermersch P, Kuiper M, Steinling M, Wolters EC, Valk J. Atrophy of medial temporal lobes on MRI in "probable" Alzheimer's disease and normal ageing: diagnostic value and neuropsychological correlates. J Neurol Neurosurg Psychiatry. 1992; 55:967–72. 10.1136/jnnp.55.10.967 1431963 PMC1015202

[31] Scheltens P, Launer LJ, Barkhof F, Weinstein HC, van Gool WA. Visual assessment of medial temporal lobe atrophy on magnetic resonance imaging: interobserver reliability. J Neurol. 1995; 242:557–60. 10.1007/BF00868807 8551316

[32] Claus JJ, Staekenborg SS, Holl DC, Roorda JJ, Schuur J, Koster P, Tielkes CE, Scheltens P. Practical use of visual medial temporal lobe atrophy cut-off scores in Alzheimer’s disease: validation in a large memory clinic population. Eur Radiol. 2017; 27:3147–55. 10.1007/s00330-016-4726-3 28083697 PMC5491609

[33] Pereira JB, Cavallin L, Spulber G, Aguilar C, Mecocci P, Vellas B, Tsolaki M, Kłoszewska I, Soininen H, Spenger C, Aarsland D, Lovestone S, Simmons A, et al, and AddNeuroMed consortium and for the Alzheimer’s Disease Neuroimaging Initiative. Influence of age, disease onset and ApoE4 on visual medial temporal lobe atrophy cut-offs. J Intern Med. 2014; 275:317–30. 10.1111/joim.12148 24118559

[34] Aziz AL, Giusiano B, Joubert S, Duprat L, Didic M, Gueriot C, Koric L, Boucraut J, Felician O, Ranjeva JP, Guedj E, Ceccaldi M. Difference in imaging biomarkers of neurodegeneration between early and late-onset amnestic Alzheimer’s disease. Neurobiol Aging. 2017; 54:22–30. 10.1016/j.neurobiolaging.2017.02.010 28314160

[35] Banerjee G, Jang H, Kim HJ, Kim ST, Kim JS, Lee JH, Im K, Kwon H, Lee JM, Na DL, Seo SW, Werring DJ. Total MRI small vessel disease burden correlates with cognitive performance, cortical atrophy, and network measures in a memory clinic population. J Alzheimers Dis. 2018; 63:1485–97. 10.3233/JAD-170943 29843234

[36] Lawrence AJ, Brookes RL, Zeestraten EA, Barrick TR, Morris RG, Markus HS. Pattern and rate of cognitive decline in cerebral small vessel disease: a prospective study. PLoS One. 2015; 10:e0135523. 10.1371/journal.pone.0135523 26273828 PMC4537104

[37] Wu B, Yao X, Lei C, Liu M, Selim MH. Enlarged perivascular spaces and small diffusion-weighted lesions in intracerebral hemorrhage. Neurology. 2015; 85:2045–52. 10.1212/WNL.0000000000002169 26546632 PMC4676754

[38] Fazekas F, Chawluk JB, Alavi A, Hurtig HI, Zimmerman RA. MR signal abnormalities at 1.5 T in Alzheimer’s dementia and normal aging. AJR Am J Roentgenol. 1987; 149:351–56. 10.2214/ajr.149.2.351 3496763

[39] Gregoire SM, Chaudhary UJ, Brown MM, Yousry TA, Kallis C, Jäger HR, Werring DJ. The microbleed anatomical rating scale (MARS): reliability of a tool to map brain microbleeds. Neurology. 2009; 73:1759–66. 10.1212/WNL.0b013e3181c34a7d 19933977

[40] Vernooij MW, van der Lugt A, Ikram MA, Wielopolski PA, Niessen WJ, Hofman A, Krestin GP, Breteler MM. Prevalence and risk factors of cerebral microbleeds: the rotterdam scan study. Neurology. 2008; 70:1208–14. 10.1212/01.wnl.0000307750.41970.d9 18378884

[41] Doubal FN, MacLullich AM, Ferguson KJ, Dennis MS, Wardlaw JM. Enlarged perivascular spaces on MRI are a feature of cerebral small vessel disease. Stroke. 2010; 41:450–54. 10.1161/STROKEAHA.109.564914 20056930

[42] Cockrell JR, Folstein MF. Mini-mental state examination (MMSE). Psychopharmacol Bull. 1988; 24:689–92. 3249771

[43] Lu J, Guo Q, Hong Z, Shi W, Lv C. Trail making test used by Chinese elderly patients with mild cognitive impairment and mild Alzheimer dementia. Chin J Clin Psychol. 2006; 14:118–21.

[44] Stroop JR. Studies of interference in serial verbal reactions. J Exp Psychol. 1935; 18:643–662. 10.1037/h0054651

[45] Lezak MD, Howieson DB, Loring DW. Neuropsychological Assessment, 4th Edn. New York: Oxford University Press; 2004.

[46] Guo Q, Zhao Q, Chen M, Ding D, Hong Z. A comparison study of mild cognitive impairment with 3 memory tests among Chinese individuals. Alzheimer Dis Assoc Disord. 2009; 23:253–59. 10.1097/WAD.0b013e3181999e92 19812468

[47] Guo Q, Hong Z, Shi W, Sun Y, Lv C. Boston naming test using by Chinese elderly, patient with mild cognitive impairment and Alzheimer's dementia. Chin Ment Health J. 2006; 20:81–85.

[48] Shin MS, Park SY, Park SR, Seol SH, Kwon JS. Clinical and empirical applications of the Rey-Osterrieth complex figure test. Nat Protoc. 2006; 1:892–99. 10.1038/nprot.2006.115 17406322

[49] Guo Q, Sun Y, Yuan J, Hong Z, Lv C. Application of eight executive tests in participants at Shanghai communities. Chin J Behav Med Sci. 2007; 16:628–31. 10.3760/CMA.J.ISSN.1005-8559.2007.07.022

[50] Guo Q, Sun Y, Yu P, Hong Z, Lv C. Norm of auditory verbal learning test in the normal aged in Chinese community. Chin J Clin Psychol. 2007; 15:132–35.

[51] Gorelick PB, Scuteri A, Black SE, Decarli C, Greenberg SM, Iadecola C, Launer LJ, Laurent S, Lopez OL, Nyenhuis D, Petersen RC, Schneider JA, Tzourio C, et al, and American Heart Association Stroke Council, and Council on Epidemiology and Prevention, and Council on Cardiovascular Nursing, and Council on Cardiovascular Radiology and Intervention, and Council on Cardiovascular Surgery and Anesthesia. Vascular contributions to cognitive impairment and dementia: a statement for healthcare professionals from the american heart association/american stroke association. Stroke. 2011; 42:2672–713. 10.1161/STR.0b013e3182299496 21778438 PMC3778669

